# Audit on High Dose Antipsychotic Treatment (HDAT) Monitoring at Rampton Hospital

**DOI:** 10.1192/bjo.2021.494

**Published:** 2021-06-18

**Authors:** Astha Das, Ian Yanson

**Affiliations:** Nottinghamshie Healthcare NHS Trust

## Abstract

**Aims:**

- High Dose Antipsychotic Treatment defined as 100% of the maximum recommended dose in British National Formulary, either as single agent or in combination.
- HDAT and poly-pharmacy may be linked to heightened mortality for psychiatric patients. The Committee on Safety of Medicines, Medicines and Healthcare Products Regulatory Agency recommended ECGs, electrolyte monitoring after each dose escalation, and 6 monthly intervals.
- The Royal College of Psychiatrists in 2006 suggested some justifiable cases of temporary poly-pharmacy with careful monitoring.
- This audit has been done in past to improve standards, especially in High Secure Setting where prescribing HDAT is a common practice
- To audit adherence to “HDAT monitoring guidelines” including regular monitoring of bloods, physical observations and ECG , done after every dose escalation plus at every 6 months.
- To monitor compliance with consent to treatment documentation including reasons of being on HDAT, documentation of physical health monitoring results

**Method:**

- All patients prescribed high dose antipsychotic (regular and as required) were identified by treating Consultants and also going through drug cards.
- One year retrospective review of haematological, ECG and physical observations were identified through Electronic notes

**Result:**

- 6 % of patients received HDAT within Rampton Hospital in 2018(12 males’ vs 6 females).
- All patients on Regular HDAT had yearly TFT done whereas only 71% had prolactin monitoring done.
- Approximately 50-60% of patients had quarterly blood monitoring including glucose, electrolytes, lipids, liver function test and full blood count.
- About 40% of patients had quarterly ECG monitoring recorded.
- 100% patients on regular HDAT had quarterly physical observation monitoring compared to 81% patients on HDAT (including PRN).
- Consent forms were completed for all patients on HDAT. 85% patients on regular HDAT has the reasons for treatment documented in the notes compared to 100% patients on HDAT (including PRN).

**Conclusion:**

- Improvement in monitoring of blood parameters and cardiac function (ECG) 40-60% as compared to 2014 audit (8% to 23%).
- Yearly monitoring of TFTs and Prolactin also appeared better (100% and 71%) which was (88% and 72% in 2014).
- Quarterly physical observations were recorded in 77% patients on regular HDAT in 2014 which improved to 100% in 2018.
- There was slight difference for those who were on PRN (77% to 81%). All prescribers informed about results and reminded of recommended guidelines
- Reaudit in 2021-22 to measure change in clinical practice in prescribing HDAT.

